# The Impact of Semaglutide on Metabolic Syndrome: A Case Report

**DOI:** 10.7759/cureus.87223

**Published:** 2025-07-03

**Authors:** Jimmy Joseph

**Affiliations:** 1 Internal Medicine, Aster DM Healthcare, Dubai, ARE

**Keywords:** diabetes, dyslipidemia, mash, metabolic syndrome, ozempic, semaglutide

## Abstract

This case describes a 44-year-old male with poorly controlled type 2 diabetes mellitus, dyslipidemia, and hypertension - hallmarks of metabolic syndrome - who demonstrated significant clinical improvement following the initiation of semaglutide, a glucagon-like peptide-1 receptor agonist (GLP-1 RA), after discontinuing sitagliptin. The patient had previously been treated with multiple oral hypoglycemic agents and basal insulin, yet continued to have inadequate glycemic and lipid control. Upon replacing sitagliptin with semaglutide, he experienced marked reductions in glycated hemoglobin, low-density lipoprotein cholesterol, and urine albumin-creatinine ratio, along with improvements in liver enzyme levels and stable renal function. This case highlights the pleiotropic benefits of GLP-1 receptor agonists and supports their role in the comprehensive management of metabolic syndrome.

## Introduction

Type 2 diabetes mellitus (T2DM) is a progressive, multifactorial disease that frequently coexists with other components of metabolic syndrome, including hypertension, dyslipidemia, central obesity, and insulin resistance. This clustering of conditions substantially elevates the risk of atherosclerotic cardiovascular disease, heart failure, and chronic kidney disease (CKD) - the leading causes of morbidity and mortality among individuals with diabetes worldwide [1,2]. Conventional glucose-lowering therapies may fall short in mitigating these risks, particularly in patients with established metabolic abnormalities. As a result, treatment paradigms are shifting from a glucose-centric focus to a more comprehensive cardiometabolic approach. Glucagon-like peptide-1 receptor agonists (GLP-1 RAs) have become central to this strategy, not only for their potent glucose-lowering properties but also for their ability to reduce body weight, improve lipid profiles, slow the progression of nephropathy, and lower the risk of cardiovascular events [3-6]. Semaglutide, a once-weekly GLP-1 RA, has demonstrated superior efficacy in reducing glycated hemoglobin (HbA1c) and body weight compared to other agents in its class. Landmark clinical trials, including SUSTAIN-6 and the STEP series, have highlighted semaglutide’s cardiovascular, renal, and hepatic benefits, such as improvements in nonalcoholic fatty liver disease (NAFLD) and reductions in albuminuria [7-9]. This case report presents real-world evidence of semaglutide’s clinical effectiveness in a middle-aged male with metabolic syndrome and poorly controlled T2DM, who achieved marked improvements in glycemic control, lipid levels, and renal parameters following treatment optimization.

## Case presentation

A 44-year-old Indian male presented for optimization of diabetes management. He had been diagnosed with T2DM, hypertension, and dyslipidemia four years earlier. He was overweight, with a BMI of 38 kg/m² and a body weight of 88 kg. His blood pressure at presentation was 146/96 mmHg. The patient denied any cardiovascular, cerebrovascular, or microvascular complications but expressed concern about suboptimal glycemic control and persistent fatigue.

At the time of presentation, his treatment regimen for diabetes included empagliflozin 10 mg twice daily, sitagliptin 50 mg twice daily, metformin 1000 mg twice daily, and insulin glargine 18 units once daily. For dyslipidemia, he was taking atorvastatin 20 mg at bedtime. His hypertension was managed with valsartan 160 mg once daily.

Baseline laboratory investigations (Table 1) indicated poor glycemic and lipid control. HbA1c was markedly elevated at 9.6% (target <7.0%), with fasting and postprandial blood glucose levels of 183 mg/dL and 265 mg/dL, respectively. His lipid profile revealed a total cholesterol level of 243 mg/dL, low-density lipoprotein (LDL) of 194 mg/dL, high-density lipoprotein (HDL) of 33 mg/dL, and triglycerides of 265 mg/dL, all outside target ranges. Atherogenic markers were also elevated, with lipoprotein(a) at 78 mg/dL and apolipoprotein B at 126 mg/dL.

**Table 1:** Baseline laboratory investigations of the patient ALT, alanine transaminase; AST, aspartate transaminase; eGFR, estimated glomerular filtration rate; GGT, gamma-glutamyl transferase; HbA1c, glycated hemoglobin; HDL, high-density lipoprotein; LDL, low-density lipoprotein; UACR, urine albumin-creatinine ratio

Test	Result	Reference range
HbA1c	9.60%	<7.0%
Fasting plasma glucose	183 mg/dL	<100 mg/dL
Postprandial blood glucose	265 mg/dL	<140 mg/dL
Total cholesterol	243 mg/dL	<200 mg/dL
LDL	194 mg/dL	<100 mg/dL
HDL	33 mg/dL	>40 mg/dL
Triglycerides	265 mg/dL	<150 mg/dL
Lipoprotein(a)	78 mg/dL	<75 mg/dL
Apolipoprotein B	126 mg/dL	<120 mg/dL
AST	76 U/L	<40 U/L
ALT	99 U/L	<40 U/L
GGT	66 U/L	<55 U/L
Serum creatinine	1.3 mg/dL	0.7-1.3 mg/dL
eGFR	58 mL/min/1.73 m²	>90 mL/min/1.73 m²
UACR	245 mg/g	<30 mg/g

Liver enzymes were raised: aspartate transaminase (AST) was 76 U/L, alanine transaminase (ALT) was 99 U/L, and gamma-glutamyl transferase was 66 U/L. Kidney function tests showed mildly elevated serum creatinine at 1.3 mg/dL, an estimated glomerular filtration rate (eGFR) of 58 mL/min/1.73 m², and a urine albumin-creatinine ratio (UACR) of 245 mg/g.

**Figure 1:**
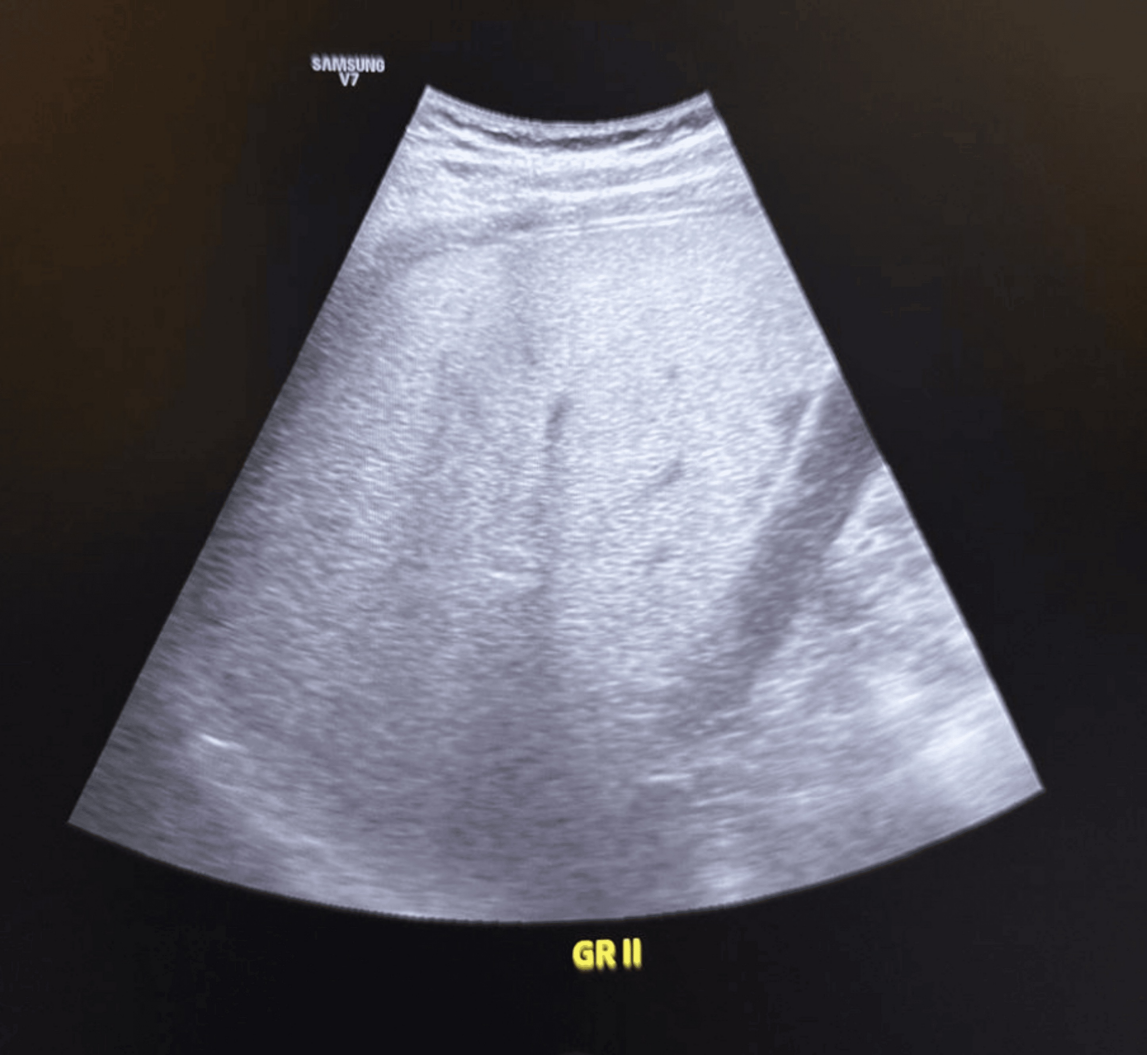
Abdominal ultrasound findings

Abdominal ultrasound revealed Grade 2 fatty liver (Figure 1).

Due to inadequate glycemic and lipid control, the patient was advised to follow a structured dietary and exercise regimen. A high-fiber, low-glycemic index diet was recommended, including vegetables, whole grains, legumes, lean protein sources, and healthy fats. He was also counseled on the importance of engaging in at least 150 minutes of moderate-intensity exercise per week.

Pharmacologic therapy was adjusted: sitagliptin was discontinued, and semaglutide (Ozempic) was initiated at a dose of 0.25 mg subcutaneously once weekly and titrated to 1 mg weekly over eight weeks. To achieve a greater reduction in LDL cholesterol, ezetimibe 10 mg was added to his statin regimen.

Follow-up investigations at three and six months (Table 2) demonstrated progressive improvement in metabolic and renal parameters. HbA1c decreased from 9.6% at baseline to 8.2% at three months and further to 7.1% at six months, approaching the target of <7.0%. Total cholesterol improved from 243 mg/dL to 198 mg/dL at three months and 176 mg/dL at six months. LDL cholesterol declined from 194 mg/dL to 160 mg/dL and then to 116 mg/dL. HDL cholesterol showed a gradual rise from 33 mg/dL to 38 mg/dL, reaching 43 mg/dL at six months, meeting the target for males. Triglyceride levels decreased from 265 mg/dL at baseline to 190 mg/dL at three months and 156 mg/dL at six months.

**Table 2:** Follow-up laboratory investigations at three and six months ALT, alanine transaminase; AST, aspartate transaminase; eGFR, estimated glomerular filtration rate; HbA1c, glycated hemoglobin; HDL, high-density lipoprotein; LDL, low-density lipoprotein; UACR, urine albumin-creatinine ratio

Parameter	At three months	At six months	Reference range
HbA1c	8.20%	7.10%	<7.0%
Total cholesterol	198 mg/dL	176 mg/dL	<200 mg/dL
LDL	160 mg/dL	116 mg/dL	<100 mg/dL
HDL	38 mg/dL	43 mg/dL	>40 mg/dL
Triglycerides	190 mg/dL	156 mg/dL	<150 mg/dL
AST	56 U/L	36 U/L	<40 U/L
ALT	78 U/L	45 U/L	<40 U/L
Creatinine	1.1 mg/dL	1.0 mg/dL	0.7-1.3 mg/dL
eGFR	52 mL/min/1.73 m²	59 mL/min/1.73 m²	>90 mL/min/1.73 m²
UACR	212 mg/g	175 mg/g	<30 mg/g

Liver function tests also showed marked improvement: AST levels dropped from 76 U/L to 56 U/L and then to 36 U/L, while ALT levels declined from 99 U/L to 78 U/L and finally to 45 U/L. Renal parameters stabilized, with serum creatinine decreasing from 1.3 mg/dL to 1.1 mg/dL and then 1.0 mg/dL. eGFR increased slightly from 52 to 59 mL/min/1.73 m². The UACR also showed a downward trend, decreasing from 245 mg/g at baseline to 212 mg/g at three months and 175 mg/g at six months, although it remained above the normal threshold.

During the follow-up period, the patient reported noticeable clinical improvements that aligned with the observed biochemical progress. He experienced better appetite control, which led to more consistent meal patterns and fewer episodes of overeating. This was accompanied by a subjective increase in energy levels, resulting in improved daily functioning and greater tolerance for physical activity. Over the six-month period, the patient achieved modest yet meaningful weight loss of approximately 4 kg, indicating improved metabolic regulation and potential cardiovascular benefit.

Importantly, there were no reported episodes of hypoglycemia, suggesting that the glycemic management regimen was both effective and safe. The patient also did not experience any gastrointestinal side effects, such as nausea, vomiting, or abdominal discomfort, that are commonly associated with some glucose-lowering therapies. The absence of adverse effects contributed to high treatment adherence and a positive overall experience, reinforcing the sustainability of the therapeutic approach.

These improvements, both objective and subjective, highlight the clinical benefits of a comprehensive diabetes management plan that incorporates pharmacologic optimization alongside active patient engagement.

## Discussion

This case highlights the substantial impact of semaglutide in a patient with uncontrolled T2DM and features of metabolic syndrome. Despite being on multiple oral agents and basal insulin, the patient’s glycemic and lipid targets remained unmet. The introduction of semaglutide resulted in marked improvements within six months, including a reduction in HbA1c from 9.6% to 7.1%, LDL cholesterol from 194 mg/dL to 116 mg/dL, and UACR from 245 mg/g to 175 mg/g.

GLP-1 RAs such as semaglutide have demonstrated efficacy in reducing major adverse cardiovascular events (MACE) and slowing the progression of diabetic nephropathy. The SUSTAIN-6 trial showed that semaglutide reduced the risk of MACE by 26% and significantly decreased the progression of albuminuria in patients with T2DM and high cardiovascular risk [4,6]. Additionally, semaglutide contributes to weight loss, improvement in hepatic steatosis, and favorable changes in lipid metabolism, key factors in the management of metabolic syndrome and NAFLD [7,9].

In this case, the patient also showed improvement in liver enzymes (AST and ALT), potentially reflecting reduced hepatic fat content. These findings are consistent with results from the STEP trials and other real-world data supporting the role of semaglutide in NAFLD. Notably, the patient did not report any adverse events such as hypoglycemia or intolerable gastrointestinal symptoms, and renal function remained stable. This supports the safe use of GLP-1 RAs even in individuals with early-stage CKD.

Overall, this case reinforces the importance of considering therapy escalation in patients with persistent metabolic derangements and supports current guideline recommendations advocating for the early use of agents with proven cardio-renal benefits in high-risk individuals [10].

## Conclusions

Semaglutide offers multifaceted metabolic benefits in patients with T2DM and metabolic syndrome, extending beyond glycemic control. This case illustrated significant improvements in HbA1c, hepatic inflammation markers, renal function, and lipid profile following the initiation of semaglutide therapy. The reduction in liver enzyme levels suggested a potential reversal of metabolic-associated steatohepatitis, while the decrease in albuminuria and stabilization of eGFR indicated renal protection. Improvements in lipid parameters also contributed to a reduction in overall cardiovascular risk.

Notably, the patient was able to safely discontinue insulin and sulfonylurea therapy, reflecting improved insulin sensitivity and a lower risk of hypoglycemia. Modest weight loss further supported these metabolic gains. This case underscores the value of early initiation of GLP-1 receptor agonists like semaglutide in high-risk individuals to promote comprehensive metabolic optimization. Clinicians should consider timely therapeutic intensification with these agents to enhance outcomes and reduce long-term complications in patients with complex metabolic disease.
